# Cellulose-based hydrogel matrix enhances antimicrobial and biofilm-inhibitory responses of palatal mesenchymal stem cells

**DOI:** 10.1007/s13205-026-04852-6

**Published:** 2026-05-15

**Authors:** Mesude Bicer, Fatma Sener, Esengül Öztürk, Özkan Fidan

**Affiliations:** Department of Bioengineering, Faculty of Life and Natural Sciences, Abdullah Gul University, Kayseri, Turkey

**Keywords:** Palatal mesenchymal stem cells, Cellulose-based hydrogel, Antimicrobial and Biofilm-Inhibitory activity, Cathelicidin (LL-37)

## Abstract

Mesenchymal stem cells (MSCs) have emerged as promising alternatives to fight drug-resistant bacterial infections. This study investigates the antibacterial activity of palatal adipose tissue-derived MSCs (PMSCs), particularly when cultured within a 3D nanofibrillar cellulose hydrogel, against four clinically relevant pathogens: *Pseudomonas aeruginosa* K6, *Staphylococcus aureus* ATCC 25,923, *Bacillus cereus* K9 and *Escherichia coli* O157:H7. This study showed that both PMSCs alone and PMSCs in 3D cellulose-based hydrogel effectively inhibited the growth of bacterial burden. Notably, PMSCs cultured in the 3D system demonstrated an excellent effect, reducing bacterial burden by up to 14 log in *E. coli* and 12 log in *P. aeruginosa* K6 at a 120 µL inoculum after 2 h of incubation. RT-PCR and immunocytochemical analyses found out a remarkable upregulation of the Cathelicidin (LL-37) in PMSCs 3D cultures compared to PMSCs. Furthermore, 3D cellulose-based hydrogel exhibited a significant biofilm-inhibitory effect, reaching a 57.65% reduction. The results demonstrated the importance of 3D cellulose-based hydrogel for treating antibiotic-resistant infections. PMSC therapy based on 3D hydrogel may therefore be offered as more effective antimicrobial agent to overcome drug-resistant bacterial infections.

## Introduction

The growing prevalence of antibiotic resistance has significantly limited the efficacy of antimicrobial therapies, thereby increasing the incidence of infections that are challenging to overcome. Of notable concern are multidrug-resistant (MDR) bacteria, which display health-threatening factors due to their capability to form biofilms that make infections more resistant to both therapeutic agents and host immune systems (Salam et al. 2023; Ahmed et al. 2024; Ho et al. 2025).

Pathogenic *E. coli* have been reported in a wide spectrum of diseases, from gastrointestinal infections to more severe systemic conditions (Cabrera-Sosa and Ochoa 2020; Pokharel et al. 2023). In case of diarrheal disease, particularly bloody diarrhea, antibiotic use is generally not recommended, due to its potential to increase the risk of hemolytic uremic syndrome (Pokharel et al. 2023). Despite this, antibiotic treatment continues to play a significant role in treating *E. coli* infections, with agents such as fluoroquinolones and trimethoprim/sulfamethoxazole frequently prescribed. However, the antimicrobial effectiveness of these agents is gradually diminishing due to increasing resistance rates, especially against sulfonamide, tetracycline and ampicillin. This raises critical concerns about MDR *E. coli* strains (Tadesse et al. 2012).

*Bacillus cereus*, another clinically relevant pathogen, exists in both vegetative cells and resilient spore forms. It is known to result in foodborne illnesses manifesting as either emetic or diarrheal conditions. Beyond gastrointestinal infections, *B. cereus* is implicated in various extraintestinal infections including pneumonia, septicemia, ocular complications, and central nervous system diseases (Jessberger et al. 2020; Sornchuer et al. 2024). The strain exhibits natural resistance to several β-lactam antibiotics (e.g., ampicillin, penicillin G and cefotaxime) primarily due to its ability to produce β-lactamases. Moreover, its robust ability to form biofilms facilitates horizontal gene transfer, which exacerbates antibiotic resistance and contributes to chronic infections (Sornchuer et al. 2022).

*Staphylococcus aureus* is among the most concerning bacterial pathogens due to its high virulence and evolving resistance to antimicrobial agents. They are related to a variety of infections ranging from mild skin conditions to life-threatening disorders (Zhu et al. 2022; Taylor and Unakal 2025). Methicillin-susceptible *S. aureus* (MSSA) infections can still be treated with β-lactam antibiotics, whereas methicillin-resistant strains (MRSA) have resulted in high mortality rates, largely due to their antibiotic resistance (Taylor and Unakal 2025). The persistence of *S. aureus* stems from their ability to develop biofilms, which act as defensive barriers that protect the bacteria from antimicrobial agents (Chow et al. 2020; Petkova et al. 2023).

*Pseudomonas aeruginosa* is another prevalent opportunistic pathogen that is associated with chronic respiratory diseases including sepsis and pneumonia (Reynolds and Kollef 2021). This pathogen is common in intensive care environments and results in a substantial number of infections (Tuon et al. 2022). These organisms, which have the potential to form biofilms, have also developed resistance mechanisms, thereby contributing to the emergence of MDR strains and complicating their response to therapeutic approaches (Ibrahim et al. 2020; Yahav et al. 2020). With the increasing burden of infections including biofilm-mediated and antibiotic resistance, advancing research and developing innovative treatment strategies has become increasingly critical.

Recently, MSCs have emerged as an interesting therapeutic candidate for the treatment of bacterial infections, due to their capability in promoting tissue regeneration and immune responses (Alcayaga-Miranda et al. 2017; Marrazzo et al. 2019; Ren et al. 2020; Castro Ramos et al. 2024). Their antimicrobial potential is mediated through direct mechanisms, which promote the secretion of antimicrobial peptides (AMPs) including hepcidin, lipocalin 2, cathelicidin-LL37, and β-defensin 2 when exposed to microbial signals (Mahlapuu et al. 2016; Sung et al. 2016; Sutton et al. 2016; Russell et al. 2020). Experimental data have shown that MSC-based treatment can significantly reduce both bacterial burden and inflammation in bacterial infection models, including *E. coli*-induced pneumonia (Krasnodembskaya et al. 2010), *Klebsiella pneumoniae*-induced sepsis (Perlee et al. 2019), and *aeruginosa*-induced peritonitis (Krasnodembskaya et al. 2012).

Despite their therapeutic potential, relevant cell numbers are often required for clinical use. However, prolonged culturing in 2D culture can lead to decreased stemness and increased genomic instability (Bara et al. 2014). These disadvantages are partly due to the lack of a physiological 3D microenvironment (Bicer et al. 2021). Unlike traditional methods, 3D cultures more closely replicate the native microenvironment of stem cells, thereby promoting the preservation of their phenotype and biological function. Previous studies have shown that 3D culture systems can significantly influence MSC behavior. For example, O’Donnell et al. expanded subcutaneous MSCs in methacrylate-gelatine hydrogels using 3D-printed bioreactors (O’Donnell et al. 2020), and this structure exhibited enhanced viability and expansion of MSCs, while Bicer et al. reported the increased proliferation of adipose-derived MSCs in nanofibrillar cellulose scaffolds compared to 2D cultures (Bicer et al. 2020). Building on these findings, the present study investigates how 3D culture of PMSCs in a cellulose-based hydrogel affects not only cell viability but also functional antibacterial and biofilm-inhibitory activities. This approach provides insight into how a physiologically relevant 3D microenvironment can potentiate MSC-mediated antimicrobial responses, highlighting its potential for therapeutic applications. These cellulose-based hydrogels are widely recognized for their biocompatibility with MSCs, supporting the viability and non-cytotoxicity to cells (Sheard et al. 2019). However, the mechanism underlying the antimicrobial and potential immunomodulatory behaviour of MSCs incorporated into this hydrogel against bacterial infections is not fully understood and needs to be investigated. Therefore, this study examined both the antibacterial and immune-related responses of Palatal MSCs cultured in cellulose-based hydrogels. It is hypothesized that Palatal MSCs embedded in this 3D scaffold will enhance the host’s antimicrobial defenses by producing LL-37 peptide. The findings from this study are expected to provide fundamental information and pave the way for the development of cellulose-based MSC therapies against bacterial infections. Such approaches are also relevant to global health priorities, particularly the United Nations Sustainable Development Goal 3 (Good Health and Well-being), which emphasizes the need for innovative strategies to combat infectious diseases and antimicrobial resistance.

## Materials and methods

### Cell culture conditions

MSCs were provided from palatal adipose tissue and were identified in accordance with the guidelines established by the International Society for Cellular Therapy (Dominici et al. 2006; Hakki et al. 2017). The human palatal MSCs (PMSCs) were kindly supplied by Prof. Dr. Sema S. Hakki at Selcuk University. No additional isolation procedures were performed in the present study. PMSCs were cultured in Dulbecco’s Modified Eagle Medium (DMEM), and supplemented with 2 mM L-glutamine, 100 U/mL penicillin, 100 µg/mL streptomycin, and 10% (*v*/*v)* heat-inactivated fetal bovine serum (FBS), with all reagents sourced from Sigma-Aldrich (Gillingham, UK). To enhance proliferation potential of PMSCs, 5 ng/mL basic fibroblast growth factor (bFGF; Peprotech, UK) was included. The cells were maintained at 37 °C and 5% CO_2_ using a BINDER APT.line™ C150 incubator. The culture medium was changed every two-three day, and subculturing was conducted once cells reached approximately 80% confluence.

### 3D culture methodology

Cellulose-based hydrogel (GrowDexT^®^**)** was purchased from UPM Biochemicals (Helsinki, Finland) and was prepared at a final concentration of 0.2% (w/v), as reported in previous studies (Sheard et al. 2019). Using 0.05% trypsin/EDTA (Sigma-Aldrich), PMSCs were enzymatically detached and then combined with a 3D GrowDexT^®^ hydrogel (final concentration of 0.2% (*w*/*v).* Cells were embedded into 3D GrowDexT^®^ hydrogel, at a density of 1 × 10^5^ cells/mL and were seeded into 24-well cell culture plates (Sheard et al. 2019). All experimental procedures were utilized using PMSCs between passages 7 and 11. To maintain experimental consistency, cells were used across all biological replicates.

### Direct bacterial killing assay

*P. aeruginosa* K6, *S. aureus* ATCC 25,923, *B. cereus* K9, and *E. coli* O157:H7 strains were cultured overnight in Luria–Bertani (LB) broth (Miller’s LB Broth, Condalab, Spain) at 37 °C with shaking at 220 rpm. The optical density of the overnight cultures was adjusted to 0.6 at 600 nm (OD_600_) in fresh LB broth. Aliquots of 40 µL and 120 µL of the adjusted bacterial cultures were separately added to wells containing 400 µL of 1 × 10⁵ PMSCs and incubated for 2 h at room temperature (RT).

Negative control groups included the same number of bacteria inoculated into (i) cell culture medium alone, (ii) cell culture medium containing the 3D material, and (iii) LB broth. As a positive control, bacterial cultures were incubated in LB broth supplemented with antibiotics: gentamicin (50 µg/mL), chloramphenicol (25 µg/mL), and kanamycin (50 µg/mL). To determine the number of viable bacterial cells, serial dilution (30 µL sample + 270 µL ddH₂O) followed by plating on LB agar was performed. Colony-forming units (CFUs) were manually counted to quantify bacterial survival (Chow et al. 2020). Each experimental condition was performed in triplicate to validate the consistency of the results.

### RNA extraction and quantitative real-time PCR (RT-qPCR) analysis

To quantify antimicrobial-peptide gene expression in PMSCs cultured in two-and three-dimensional formats, cells were seeded at 1 × 10^5^ cells/mL into 24-well culture plates and incubated for 24 h at 37 °C in a humidified incubator with 5% CO_2_. Parallel negative-control wells were maintained under identical conditions. For co-culture experiments with four different pathogens mentioned above, suspensions containing 40 µL and 120 µL of adjusted bacterial cultures were separately added to the respective wells, followed by a 2-hour incubation at 37 °C, 5% CO_2_. Unless otherwise stated, antibiotic-free culture medium was employed throughout the study.

Total RNA was isolated using the TransZol reagent (ET101-01; Transgen Biotech) in accordance with the manufacturer’s instructions. RNA purity and concentration were assessed via NanoDrop ND-1000 spectrophotometry. Subequently, 1 µg of total RNA was reverse-transcribed into complementary DNA (cDNA) using the EasyScript First-Strand cDNA Synthesis Kit (AE301, Transgen Biotech). Quantitative PCR assays were performed using gene-specific primers designed through Primer-BLAST (NCBI), with final primer concentrations of 100 nM the forward and 200 nM for the reverse oligonucleotide. Amplification was carried out using TransStart Green qPCR Supermix (AQ101), and fluorescence signals were recorded for the expression analysis. Gene expression levels were normalized to GAPDH as the internal reference gene, and fold changes were computed using the comparative 2^− ΔΔCt^ -method relative to untreated controls (Livak and Schmittgen 2001). The ΔCt values were obtained by subtracting the Ct of the housekeeping gene from that of the target gene. The oligonucleotide sequences used for each target are listed in Table 1 (Chow et al. 2020).

**Table 1:** The primer sequences designed for the selected genes

Gene	Amplicon size (bp)	Forward Primer ve Reverse Primer (5’ → 3’)
GAPDH:	66	Fwd 5’-AGCCACATCGCTCAGACAC Rev 5’-GCCCAATACGACCAAATCC
Beta Defensin2 (hBD2):	255	Fwd 5’-CCAGCCATCAGCCATGAGGG Rev 5’-GGAGCCCTTTCTGAATCCGC
Cathelicidin (LL37):	276	Fwd 5’-GAAGACCCAAAGGAATGGCC. Rev 5’-CAGAGCCCAGAAGCCTGAGC
Surfactant Protein D (SPD):	114	Fwd 5’-ACAAAAAGAAACCTGCCATGCT Rev 5’-TGGGCATTGTTCTGTGGGAG
Hepcidin:	93	Fwd 5’-CCCACAACAGACGGGACAAC. Rev 5’-CTCCTTCGCCTCTGGAACAT
Lipocalin (LCN2):	71	Fwd 5’-GGAGCTGACTTCGGAACTAAAGG Rev 5’-TGTGGTTTTCAGGGAGGCC

### Immunocytochemical detection of antimicrobial peptides

PMSCs cultured on glass coverslips (2-D and 3-D formats) were fixed for 10 min with 4% Paraformaldehyde (PFA), rinsed in phosphate-buffered saline (PBS), and permeabilized with 0.1% (v/v) Triton X-100 to facilitate intracellular antibody access. To block non-specific binding, samples were incubated with 5% (v/v) normal donkey serum. Cells were then incubated with the appropriate primary antibody, selected based on prior RT-qPCR results indicating significant upregulation of AMPs. For cathelicidin detection, coverslips were incubated for 1 h at room temperature with the Anti-Cathelicidin antibody (CAMP; FINETEST, FNab01241) diluted 1:100 (Chow et al. 2020). Following extensive PBS washes, specimens were treated for 60 min with an Alexa Fluor 555 Donkey anti-rabbit IgG secondary antibody (BioLegend, 406412) at 1:300. Nuclear counterstaining was conducted with 4′,6-diamidino-2-phenylindole (DAPI) for 20 min. Fluorescence images were acquired using a confocal laser-scanning microscope under identical settings for all samples to ensure consistency in comparative analysis.

### Biofilm formation assay

Biofilm assay was performed based on the standard microplate dish protocol (O’Toole 2011). *P. aeruginosa* K6 strain was grown overnight in LB broth at 37 °C with shaking at 220 rpm. The overnight culture was then diluted 1:100 in fresh LB broth to an OD_600_ of 0.6, and 200 µL of the adjusted bacterial suspension was added in triplicate to each well of a sterile 96-well cell culture plate (SPL Life Sciences). The plate was incubated under static conditions at 37 °C for 24 h to allow biofilm formation. After 24 h, 100 µL of the 1 × 10⁵ PMSCs were added to the wells containing bacterial cultures. For the positive control, antibiotics (Kanamycin [50 µg/mL], Chloramphenicol [25 µg/mL] and Gentamycin [200 µg/mL]) were added at the same volume. For the negative control group, 100 µL antibiotic-free cell culture medium was added to the bacterial cultures. After 72 h of incubation, planktonic bacteria were removed, and the wells were gently washed with PBS and air-dried. Then, biofilm cells were stained with 125 µL 0.1% crystal violet dissolved in water for 15 min. Excess stain was gently washed with PBS, and plate was air-dried. The retained crystal violet, representing biofilm biomass, was solubilized with 125 µL of 95% ethanol, and absorbance was measured at 570 nm (OD_570_) using a microplate reader to quantify the biofilm.

### Biofilm disruption assay

*Pseudomonas aeruginosa* K6 strain was cultured overnight in LB broth at 37 °C with shaking at 220 rpm. The culture was diluted 1:100 in fresh LB broth to reach an OD_600_ of 0.6, and 200 µL of the suspension was dispensed, in triplicate, into each well of a sterile 96-well plate (SPL Life Sciences). The plate was incubated under static conditions at 37 °C for 72 h. After 72 h, planktonic bacteria were removed, and the wells were gently washed with PBS. Then, 200 µL of 1 × 10⁵ PMSCs were added to the wells containing the bacterial cultures, and the plates were incubated under static conditions at 37 °C for 24 h. For the control group, only cell culture medium was added. Following incubation, planktonic cells were again removed, the wells were washed with PBS, and the plates were air-dried. Biofilm biomass was stained with 125 µL of 0.1% (w/v) crystal violet for 15 min. Excess stain was rinsed off with PBS, and the plates were air-dried. Bound crystal violet was solubilized with 125 µL of 95% ethanol, and absorbance was measured at 570 nm (OD_570_) using a microplate reader to quantify the biofilm. The killing percentage was calculated based on the absorbance values using the following formula: Killing % = ((Control − Treatment) / Control) × 100.

### Statistical analysis

For pairwise comparisons involving two independent experimental conditions, the Mann-Whitney U test was employed due to its nonparametric nature. For comparisons involving more than two groups, one-way analysis of variance (ANOVA) was performed, followed by Tukey’s post hoc test to determine statistically significant differences between group means. A significant interaction term (*P* ≤ 0.05) was interpreted as indicative of synergism (Slinker 1998). Synergy was operationally defined as a significant enhancement in antibacterial activity (mean bacterial killing efficiency) observed in groups receiving antibiotics or PMSCs. All statistical procedures were executed using GraphPad Prism version 8 (GraphPad Software, La Jolla, CA, USA). The following significance thresholds were applied: *P* ≤ 0.05 (*), *P* ≤ 0.01(**), *P* ≤ 0.001 (***), *P* ≤ 0.0001 (****).

## Results

### In vitro antibacterial activity of PMSCs

**Fig. 1:**
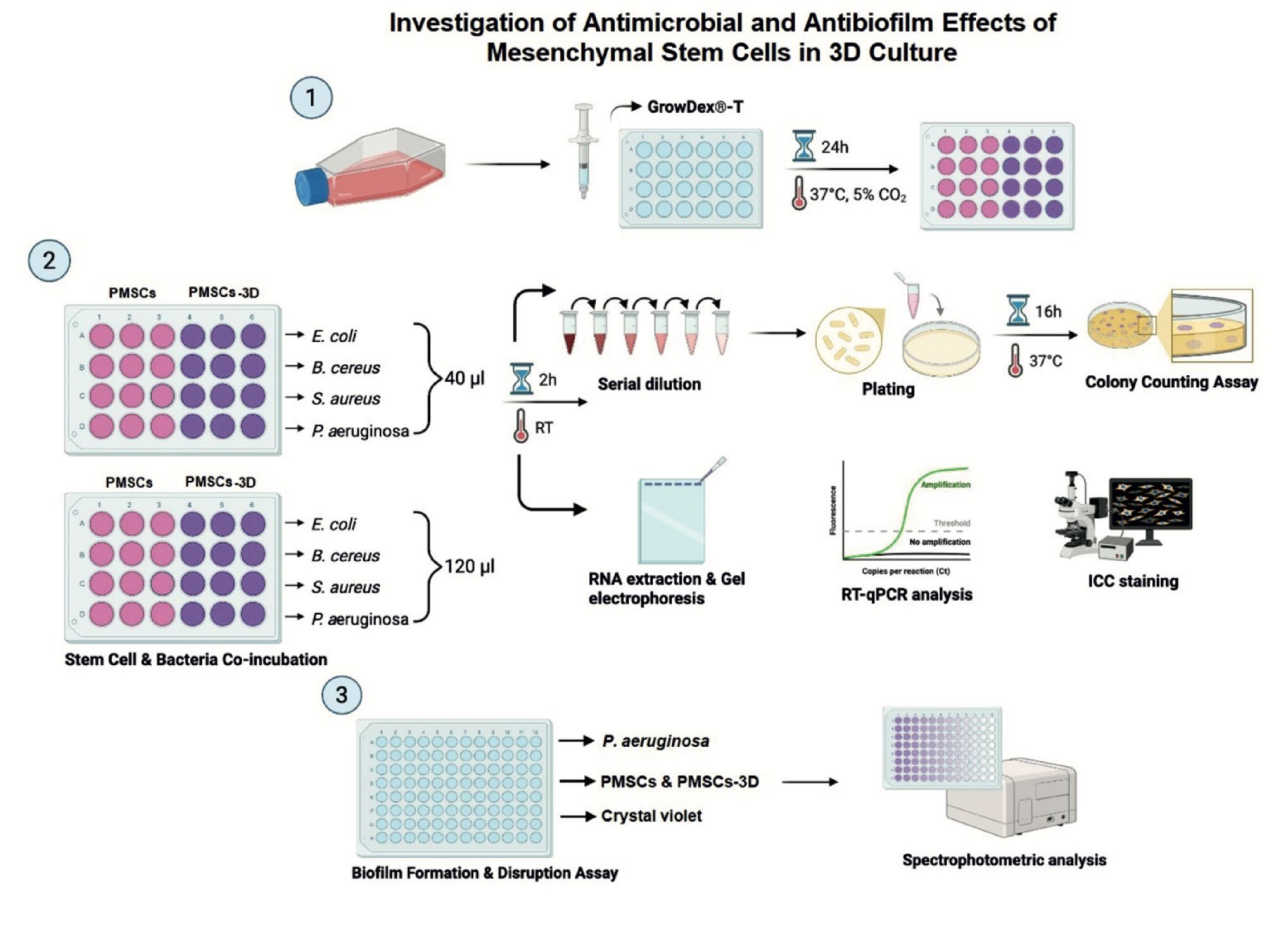
Schematic representation of the in vitro antimicrobial and antibiofilm assays. PMSCs were cultured in a 3D system, and the antibacterial effects of both PMSCs and PMSCs-3D groups were evaluated against four different pathogens: (i) *E. coli* O157:H7, (ii) *B. cereus* K9, (iii) *S. aureus* ATCC 25,923, and (iv) *P. aeruginosa* K6. The pathogens were co-incubated with PMSCs at room temperature (RT). The results were evaluated using CFU counting, RNA extraction, RT-qPCR, and ICC staining. Biofilm formation and disruption were assessed using a crystal violet assay, and the results were quantified by spectrophotometric analysis (for detailed information see materials and methods section)

**Fig. 2:**
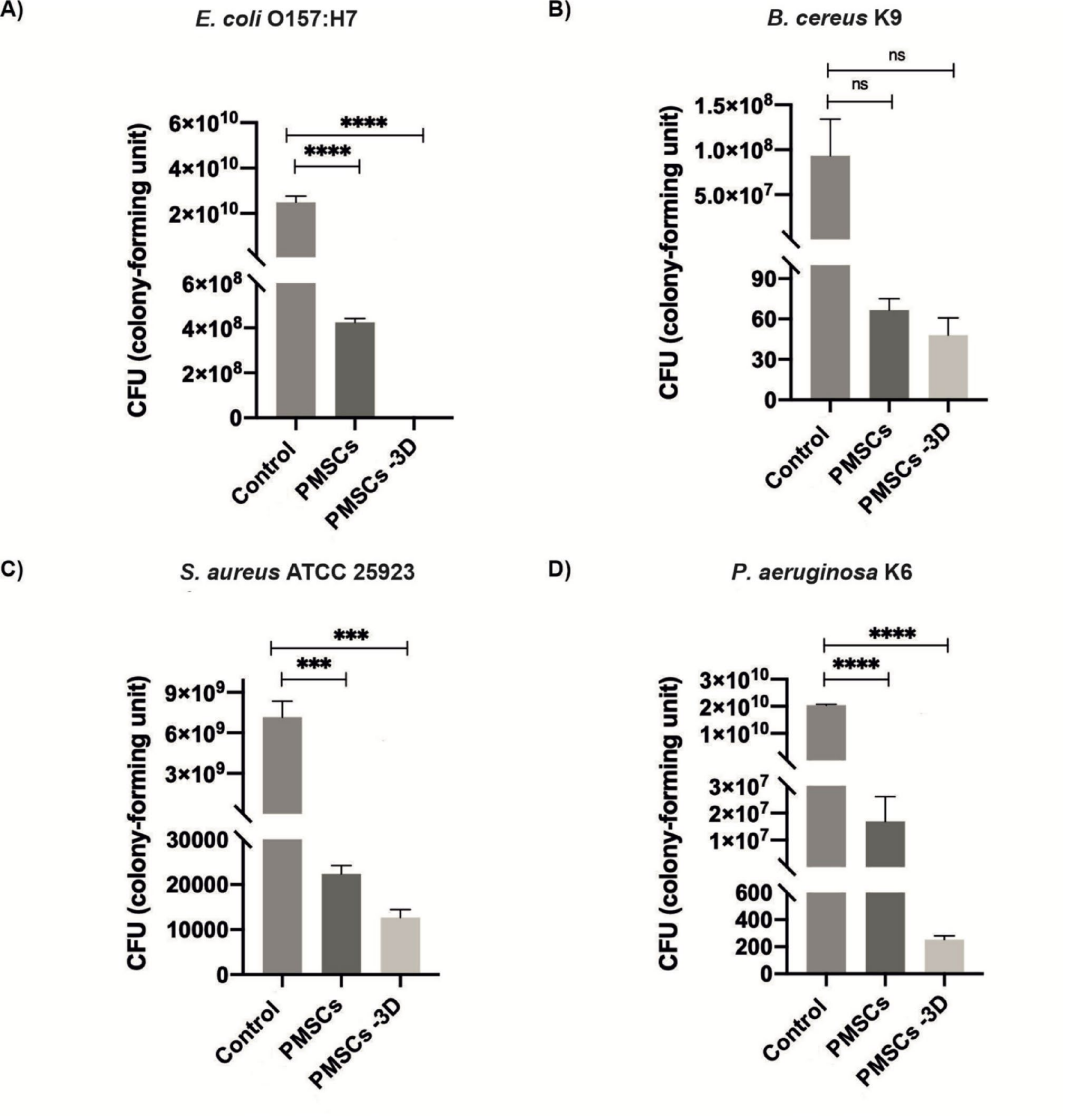
Antibacterial effects of PMSCs and PMSCs-3D with the inoculum volume of 40 µL against the bacterial strains **A** *E. coli* O157:H7, **B** *B. cereus* K9, **C** *S. aureus* ATCC 25,923, and **D** *P. aeruginosa* K6. Control groups consisted of bacteria incubated in cell culture medium without treatment. Data represent mean values from triplicate experiments (*n* = 3 representative images for each group)

**Fig. 3:**
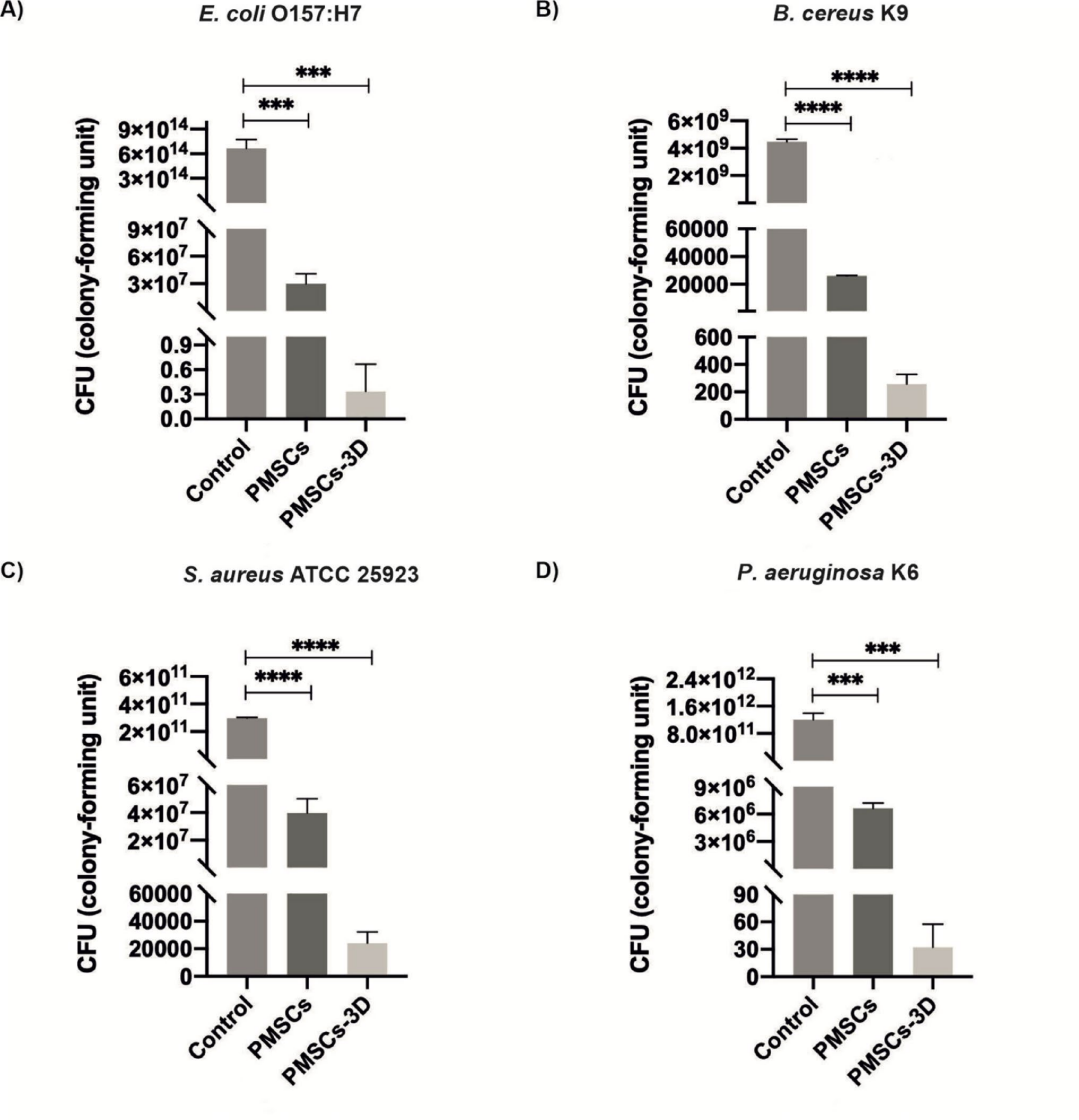
Antibacterial effects of PMSCs and PMSCs-3D with the inoculum volume of 120 µL against the bacterial strains **A** *E. coli* O157:H7, **B** *B. cereus* K9, **C** *S. aureus* ATCC 25,923, and **D** *P. aeruginosa* K6. Control groups consisted of bacteria incubated in cell culture medium without treatment. The data demonstrate the enhanced antibacterial performance of PMSCs and PMSCs-3D at higher inoculum volume, (*n* = 3 representative images for each group)

The antibacterial potential of PMSCs was initially evaluated through an in vitro bacterial killing assay targeting four different pathogens mentioned above. A 40 µL inoculum of each bacterial strain was co-cultured with viable PMSCs in triplicate (Fig. 1). This exposure resulted in a pronounced antibacterial response, with a 2-log reduction in *E. coli* O157:H7, a 7-log reduction in *B. cereus* K9, a greater than 5-log reduction in *S. aureus* ATCC 25,923, and a 3-log reduction in *P. aeruginosa* K6, compared to the negative control group containing only pathogens (Fig. 2). To evaluate the impact of increased bacterial inoculum volume on the antibacterial activity of PMSCs, the same experimental protocol was repeated using a 120 µL inoculum. Under these conditions, PMSCs induced a 7-log reduction in *E. coli* O157:H7, a 5-log reduction in *B. cereus* K9, a 4-log reduction in *S. aureus* ATCC 25,923, and a 6-log reduction in and *P. aeruginosa* K6 (Fig. 3).

### Improved antibacterial activity of PMSCs in 3D cellulose-based hydrogels

To investigate whether PMSCs cultured within the 3D platform exhibit direct antibacterial effects against the pathogens, the same experimental conditions were applied using a 3D GrowDexT^®^ hydrogel model with bacterial inocula of 40 and 120 µL, performed in triplicate (Fig. 1). Among all tested pathogens, *E. coli* O157:H7 demonstrated the most pronounced susceptibility, exhibiting complete growth inhibition (0.3 CFU) following treatment with PMSCs-3D (Fig. 2A). On the other hand, *B. cereus* K9 showed a 7-log reduction in bacterial growth for both PMSCs, and PMSCs-3D group (Fig. 2B). In addition, 5-log reduction was observed against to *S. aureus* ATCC 25,923 in the treatment with PMSC-3D group, showing only a slight quantitative difference between PMSCs (~ 20000 CFU) and PMSC-3D (~ 10000 CFU) (Fig. 2C). For *P. aeruginosa* K6, PMSCs-3D treatment resulted in a significant 8-log reduction in bacterial growth and statistical analysis revealed a significant difference between the antibacterial activity of PMSCs and PMSC-3D group, with a 5-log reduction in the bacterial count (Fig. 2D). At a higher inoculum volume of 120 µL, *E. coli* O157:H7 exhibited nearly complete growth inhibition (0.3 CFU), corresponding to a 14-log reduction compared to the control, and a 7-log difference was observed between the PMSCs and PMSCs-3D groups (Fig. 3A). For *B. cereus* K9, the treatment resulted in a 7-log reduction in bacterial count compared to its respective control group, while there was only 2-log reduction difference compared to the PMSCs group (Fig. 3B). Likewise, 7-log inhibition and 3-log inhibition was observed in *S. aureus* ATCC 25,923 after PMSC-3D and PMSCs treatments compared to the control group, respectively (Fig. 3C). Lastly, *P. aeruginosa* K6 exhibited 11-log reduction compared to its control group, while there was a 5-log reduction difference between PMSCs and PMSC-3D groups (Fig. 3D).

### Enhanced LL-37 expression in 3D-cultured PMSCs

**Fig. 4:**
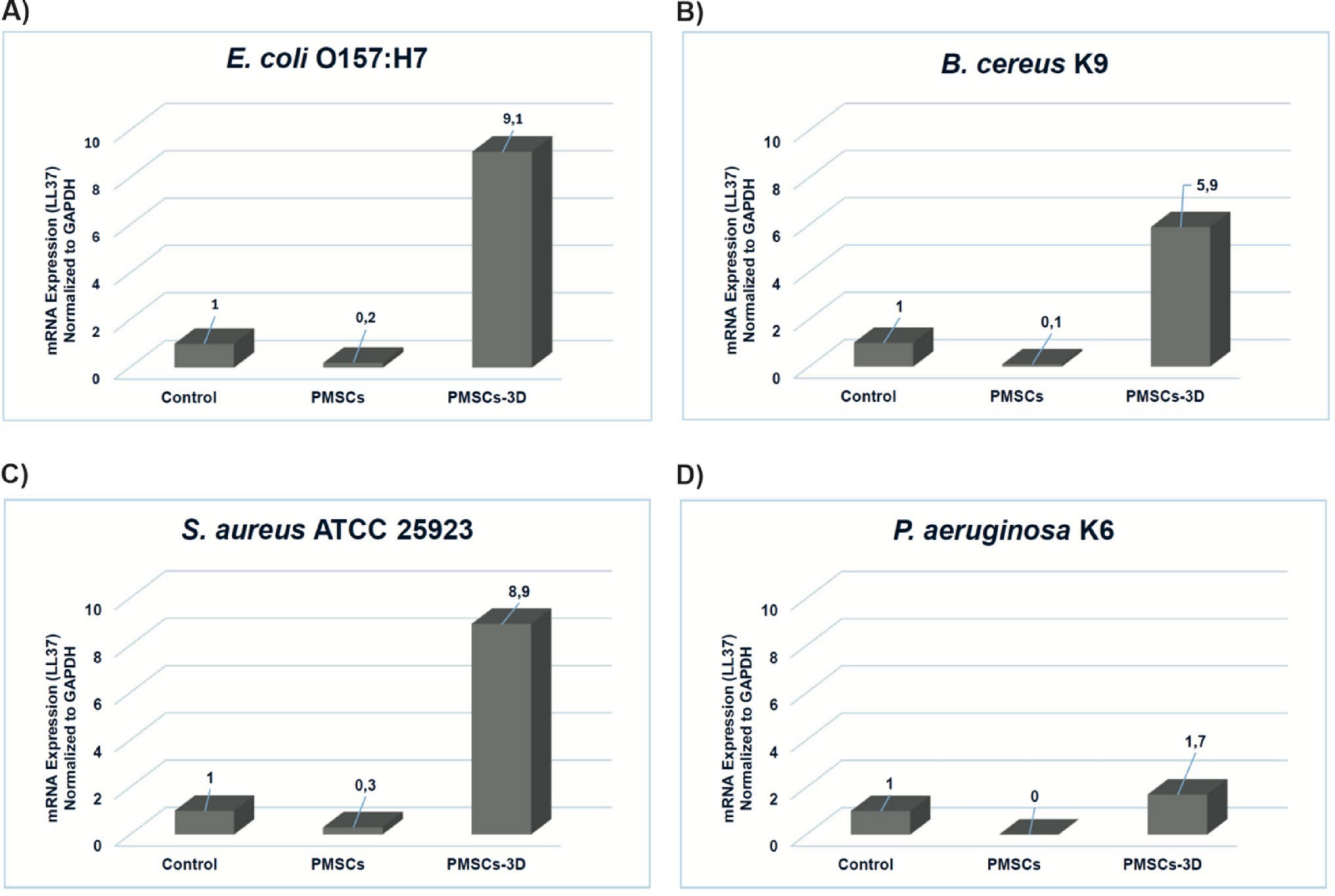
Impact of 3D hydrogel on LL-37 mRNA expression in PMSCs exposed to bacteria (40 µL inoculum volume), as assessed by RT-qPCR. The y-axis shows the fold change in gene expression relative to untreated controls and normalized to GAPDH, calculated using the ddCT method. 3D-cultured PMSCs exhibited substantially greater induction of LL-37 compared to 2D monolayers against all bacteria.** A** Quantitative representation of LL-37 expression, levels against *E. coli* O157:H7. ** B** Quantitative representation of LL-37 expression, levels against *B. cereus* K9. ** C** Quantitative representation of LL-37 expression, levels against *S. aureus* ATCC 25,923. ** D** Quantitative representation of LL-37 expression, levels against *P. aeruginosa* K6. Data points represent the mean of three technical replicates of PMSCs

**Fig. 5:**
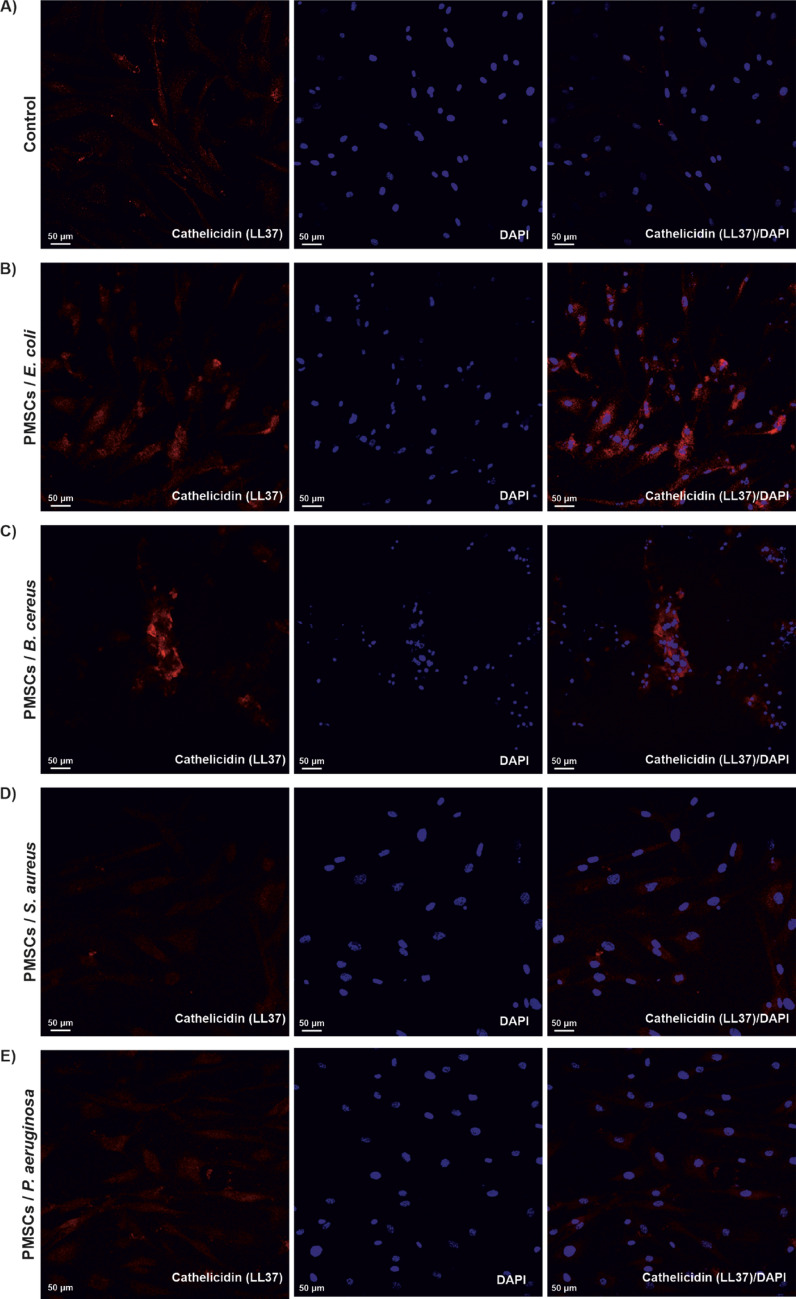
Detection of LL37 expression in PMSCs cultured in 2D by immunocytochemistry. Representative confocal images of PMSCs exposure to bacteria (40 µl inoculum volume), immunostained with antimicrobial peptide antibody, anti-LL37 antibody. Specific binding of the antibody to intracellular LL-37 is visualized in red fluorescence, while nuclear staining with DAPI appears in blue. ** A** Control, unstimulated pathogen, ** B** *E. coli* O157:H7, ** C** *B. cereus* K9, ** D** *S. aureus* ATCC 25,923, ** E** *P. aeruginosa* K6. Images were captured using a 20x objective under identical laser settings. Scale bar: 50 μm. (*n* = 3 representative images for each group).

To evaluate the transcriptional activation of AMPs in response to four different pathogens mentioned above, PMSCs were cultured under both 2D and 3D culture conditions. The expression of five AMP-related genes - cathelicidin LL-37, hepcidin, lipocalin (LcN), surfactant protein D (SPD) and human beta defensin (hBD2) - was quantified via reverse transcription quantitative polymerase chain reaction (RT-qPCR), following protocols adapted from Chow et al. (2020). Gene expression levels were normalized to GAPDH and analyzed using the comparative 2^− ΔΔCt^ -method. PMSCs maintained under unstimulated conditions without pathogen exposure did not exhibit detectable expression of the targeted AMPs. In contrast, exposure to these pathogens mentioned above led to a notable transcriptional upregulation of LL-37 gene, particularly within pathogen-stimulated samples grown in 3D platform (as depicted in Fig. 4). Among the panel of evaluated peptides, LL-37 was the only AMP demonstrating significant mRNA induction upon microbial challenge in 3D-cultured PMSCs (Fig. 4). As illustrated in Fig. 4 (40 µL bacterial inoculum), PMSCs embedded in the 3D GrowDexT^®^ hydrogel matrix, indicating that a 3D microenvironment exhibited substantially greater induction of LL-37 compared to 2D monolayers against all tested pathogens. The enhancement was most pronounced against *S. aureus* ATCC 25,923 and *E. coli* O157:H7, with a significant elevation observed across other pathogens. This 3D-mediated amplification was further intensified at higher bacterial loads. Figure 5 (120 µL inoculum) demonstrated a dose-dependent increase in LL-37 upregulation in 3D cultures, with the magnitude of induction significantly exceeding 40 µL results across all pathogens. Once again, *S. aureus* ATCC 25,923 and *E. coli* O157:H7 elicited the strongest differential response, while *P. aeruginosa* K6 exhibited the low level of expression compared to control and PMSCs. No measurable gene expression was detected for hepcidin, lipocalin (LcN), surfactant protein D (SPD), beta defensin (hBD2) under any experimental condition. Based on these findings, LL-37 was selected for downstream analyses to assess peptide-level functionality.

### Intracellular expression of LL-37 by PMSCs cultured in 3D hydrogel matrices

**Fig. 6:**
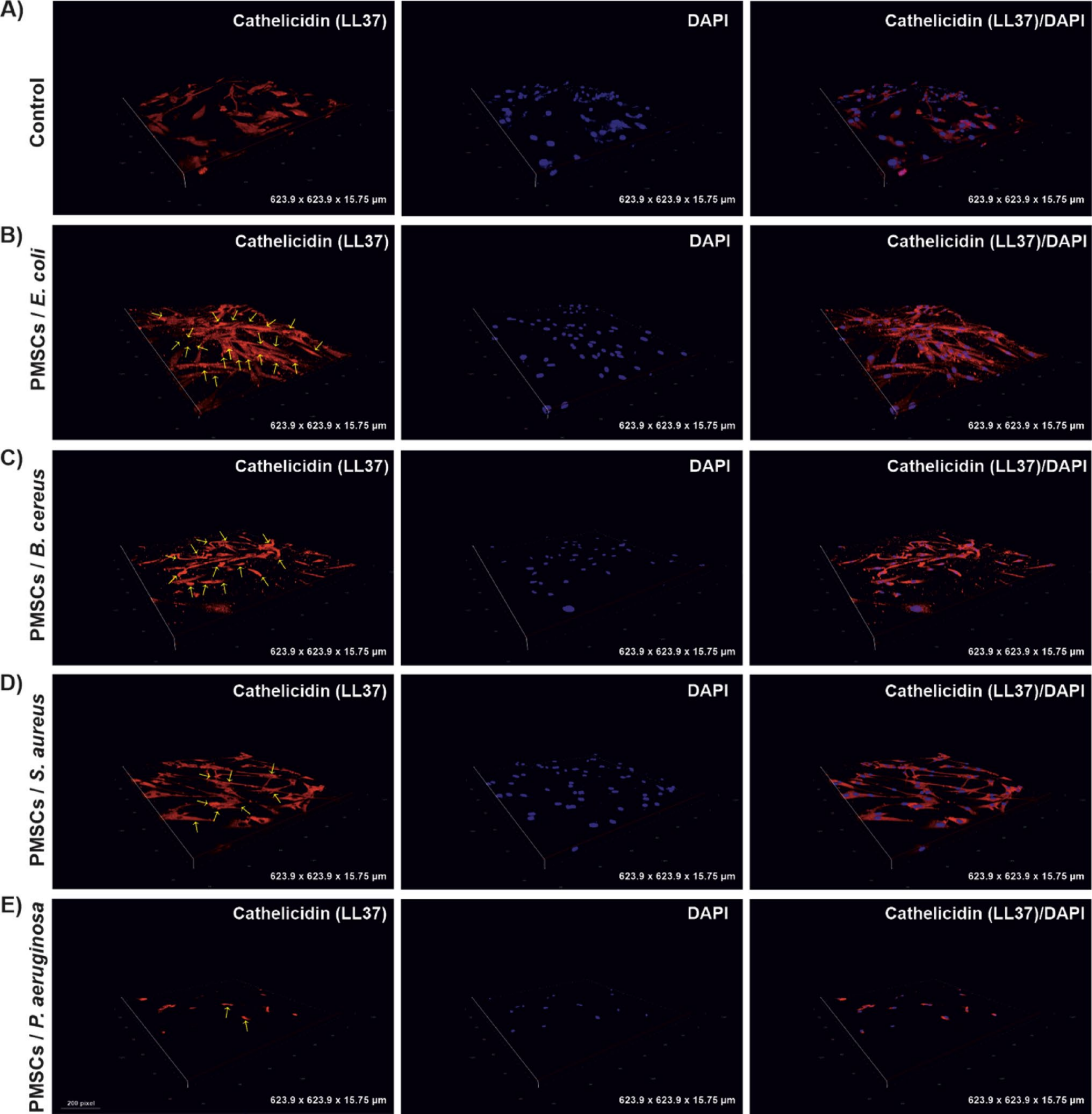
Detection of LL37 expression in PMSCs cultured in 3D by immunocytochemistry. Representative confocal images of PMSCs exposure to bacteria (40 µl inoculum volume), immunostained with antimicrobial peptide antibody, anti-LL37 antibody. Specific binding of the antibody to intracellular LL-37 is visualized in red fluorescence, while nuclear staining with DAPI appears in blue. ** A** Control, unstimulated pathogen, ** B** *E. coli* O157:H7, ** C** *B. cereus* K9, ** D** *S. aureus* ATCC 25,923, ** E** *P. aeruginosa* K6. Confocal fluorescence images of PMSCs-3D were captured using a 20× objective. 3D reconstruction of confocal Z-stack images covering a volume of 623.9 × 623.9 × 15.75 μm (0.624 μm/pixel in XY; 0.25 μm Z-step; 64 slides). Scale bar: 50 μm. . (*n* = 3 representative images for each group)

**Fig. 7:**
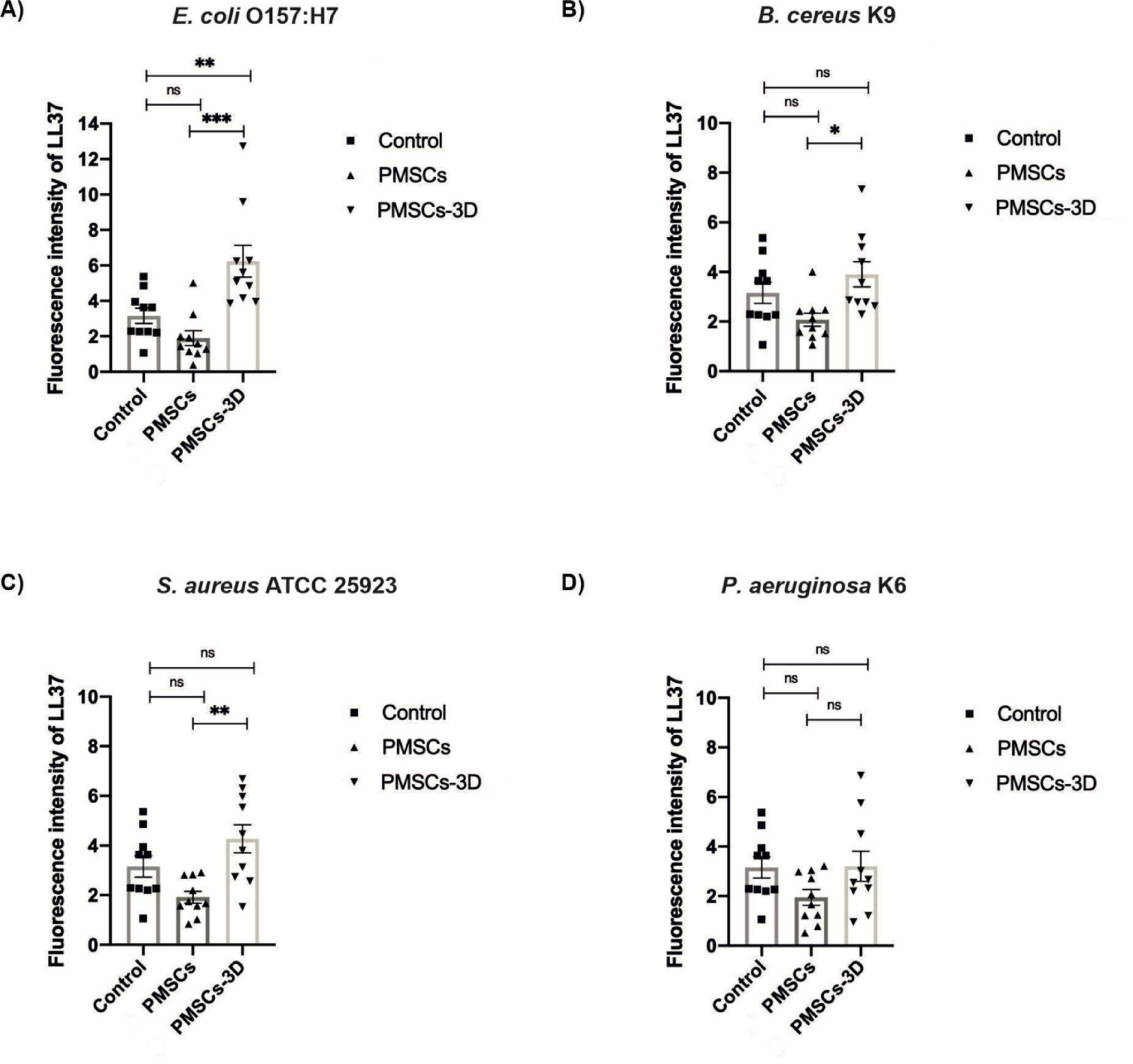
Quantification of mean fluorescence intensity for LL-37 in PMSCs cultured in 2D and 3D culture. Quantitative representation of mean fluorescence intensity for LL-37 expression, levels against bacteria (40 µl inoculum volume). ** A** *E. coli* O157:H7, ** B** *B. cereus* K9, ** C** *S. aureus* ATCC 25,923, ** D** *P. aeruginosa* K6. The y-axis represents mean fluorescence intensity of three groups including control without bacterial pathogens, PMSCs and PMSCs-3D. Data are representative of three experiments conducted with PMSCs. (*n* = 3 representative images for each group)

Consistent with earlier findings demonstrating the capacity of human MSCs to express AMPs including LL-37 (Krasnodembskaya et al. 2010; Alcayaga-Miranda et al. 2017; Chow et al. 2020), we performed immunocytochemical analysis to validate LL-37 transcriptional activation observed in RT-PCR assays. Following bacterial stimulation, PMSCs cultured in both 2D and 3D platforms were stained with an anti-LL37 antibody to assess the intracellular peptide localization. Using immunocytochemistry staining with an anti-LL37 antibody, we confirmed that PMSCs actively expressed LL-37 at the intracellular level. Confocal fluorescence imaging revealed robust intracellular expression of LL-37 in both models, with visibly stronger fluorescence intensity in the 3D hydrogel (Fig. 6) compared to 2D monolayer (Fig. 5). Nuclear counterstaining was performed using DAPI, allowing normalization of LL-37 signal intensity. Quantitative analysis of mean fluorescence intensity (MFI) confirmed enhanced LL-37 expression following bacterial exposure, particularly in the 3D-cultured PMSCs (Fig. 7). As illustrated in Fig. 7 (40 µL bacterial inoculum), 3D-cultured PMSCs exhibited substantially greater induction of LL-37 compared to 2D monolayers against all tested pathogens, except for *P. aeruginosa* K6. The enhancement was most pronounced against *E.coli* O157:H7 (*p* ≤ 0.01(**), with statistically significant elevation observed across other pathogens. These findings support the gene-level data and further suggest that 3D microenvironments not only maintain but potentially enhance the functional expression of AMPs such as LL-37 in response to bacterial pathogens.

### PMSCs cultured within the 3D platform prevents the biofilm formation

**Fig. 8:**
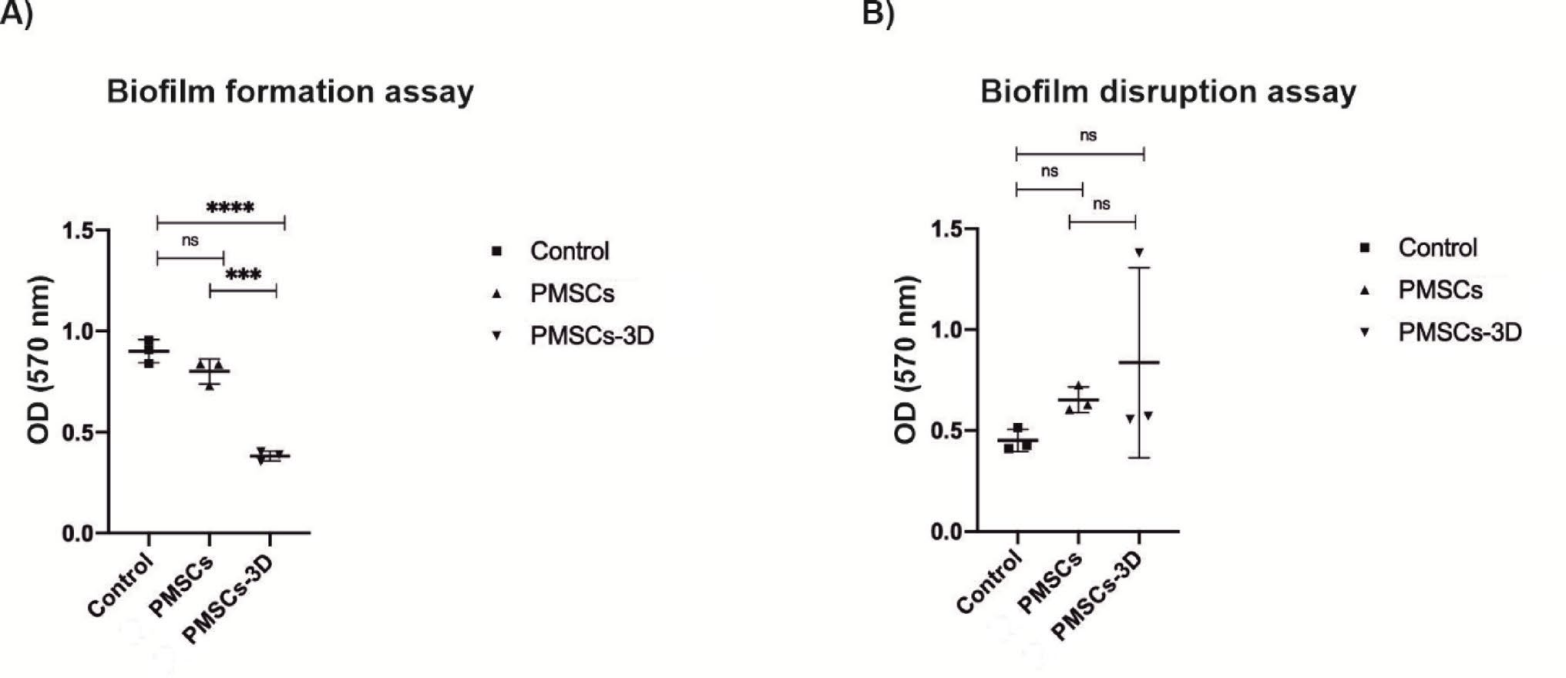
The biofilm formation and disruption assay using *P. aeruginosa* K6. ** A** The effect of PMSCs and PMSCs-3D to inhibit significantly biofilm formation, ** B** The effect of PMSCs and PMSCs-3D to inhibit significantly biofilm disruption. The experimental groups were not successful to disrupt the biofilm formed by *P. aeruginosa* K6. The results were quantified by crystal violet staining and OD_570_ measurements. (*n* = 3 representative images for each group)

A biofilm formation and disruption assay were performed using *P. aeruginosa* K6, as it exhibited the highest biofilm-forming capacity among all tested pathogens. In the experimental groups, PMSCs and PMSC-3D treatments were tested. Notably, it was observed that biofilm formation was prevented with a killing percentage of 57.60% in the PMSC-3D group (****P ≤ 0.0001), while PMSC exhibited only 11.09% reduction, which was not statistically significant. On the other hand, neither PMSCs nor PMSC-3D was effective in disrupting preformed biofilms (Fig. 8). The absorbance at 570 nm (OD₅₇₀_nm_) was measured as 0.45 for the control group, 0.65 for the PMSCs group, and 0.53 for the PMSCs-3D group.

## Discussion

Prevalent resistance to antibiotics of pathogenic bacteria increases worldwide, contributing to a significant number of deaths annually. Due to this major public health concern, there is an urgent need for the development of novel antibacterial agents (Urban-Chmiel et al. 2022). In this study, antibacterial activities of PMSCs and PMSCs cultured within 3D platform were investigated against pathogen strains mentioned above at two different inoculum volumes (40 µL and 120 µL). *E. coli* is one of the prevalent bacterial species and causes extraintestinal diseases including urinary tract infections such as pyelonephritis and acute cystitis (Arbab et al. 2022). Furthermore, *B. cereus* can exist in different environments, form spores, adapt to changing conditions, and produce harmful toxins. These features render the pathogen as a potential health hazard deserving considerable attention. *B. cereus* causes food-borne diseases and is also a causative agent of non-gastrointestinal infections. *S. aureus* is responsible for pneumonia, respiratory tract infections, cardiovascular infectious, and nosocomial bacteremia (Cheung et al. 2021; Jovanovic et al. 2021). Lastly, it was reported that treatment efficacy of antimicrobials against to *P. aeruginosa* infectious decreased recently and it is a challenging problem due to its increasing resistance to antibiotics and its involvement in clinical conditions such as pneumonia, sepsis, and urinary tract infections (de Sousa et al. 2021). Our results indicated that both PMSCs and PMSCs-3D groups effectively fight against all tested pathogens. Notably, bacterial growth was significantly inhibited in PMSCs-3D groups, with the most pronounced effect observed against *E. coli* O157:H7 at both inoculum volumes (Fig. 2 for 40 µL and Fig. 3 for 120 µL). At 40 µL, bacterial growth in the PMSCs-treated samples for *S. aureus* ATCC 25,923, *E. coli* O157:H7, *P. aeruginosa* K6, and *B. cereus* K9 was reduced by approximately 4-log, 8-log, 5-log, and 3-log compared to negative control group (containing only cell culture medium and bacteria), respectively (Fig. 2). Similarly, the PMSCs-3D group demonstrated reductions of 10-log, 8-log, 5-log, and 8-log for the same pathogens. At 120 µL inoculum, bacterial growth was reduced by 7-log, 5-log, 4-log, and 6-log in the PMSCs group, while PMSCs-3D yielded reductions of 14-log, 7-log, 7-log, and 12-log, respectively (Fig. 3). These findings suggest a dose-dependent antibacterial effect, with PMSCs-3D indicating superior effectiveness across all experimental conditions. In previous studies, bone-marrow MSCs (BM-MSCs) and adipose-derived stromal/stem cells (ACSs) had a strong antibacterial effect on *S. aureus* ATCC 25,923 (with a killing percentage of 71% and 75%, respectively), which aligns with our observations (Yagi et al. 2020).

However, it is important to acknowledge the limitations of the present study. The experiments were conducted using an in vitro model, which does not fully mimic the complexity of the in vivo microenvironment such as immune system interactions and tissue architecture. Furthermore, only a restricted number of bacterial strains was used in the study, and long-term effects of the pathogens were not evaluated. Therefore, further in vivo studies and clinical investigations are necessary to validate these findings and to better understand the underlying mechanisms of action.

MSCs have been reported to enhance host immune response by enhancing the phagocytic capacity of immune cells and dampening inflammation, which facilitates the clearance of bacterial pathogens (Mei et al. 2010; Brandau et al. 2014). In a complementary manner, antimicrobial peptides (AMPs), such as LL-37 and β-defensin-2, not only exert direct bactericidal effects but also contribute to immune regulation, highlighting a coordinated role in both pathogen elimination and modulation of host immunity (Li et al. 2023). Based on these insights, the present study examined whether PMSCs exhibit antimicrobial activity through similar peptide-based mechanisms. The results demonstrated that, for all bacterial strains analyzed, PMSCs grown in a 3D cellulose-based hydrogel displayed a significantly higher level of LL-37 gene expression than those maintained in traditional culture conditions (Fig. 4A-D). The highest level of LL-37 expression was observed in response to *E. coli* O157:H7 and *S. aureus* ATCC 25,923. The enhanced LL-37 response to *E. coli* may be linked to increased TLR-mediated signaling induced by Gram-negative bacterial components such as LPS, in line with previous studies highlighting the role of pathogen-associated molecular patterns (PAMPs) recognition in regulating antimicrobial peptide expression in MSCs (Yagi et al. 2020). Conversely, no detectable expression of other antimicrobial peptides (e.g., hepcidin, lipocalin, surfactant protein D and β-defensin-2) was exhibited. These findings are consistent with previous study indicating that LL-37 is expressed intracellularly in MSCs when exposed to bacterial infections (Chow et al. 2020). Furthermore, a statistical difference was stated between experimental and control groups, indicating that PMSCs embedded within 3D hydrogels possess superior antibacterial potential. This enhanced response is likely attributable to the supportive role of the 3D hydrogel in facilitating LL-37 gene expression, particularly against *E. coli* O157:H7, *B. cereus* K9, *S. aureus* ATCC 25,923 (Fig. 6B-D). 3D-cultured PMSCs exhibited the enhanced antibacterial and anti-biofilm activity, which may be attributed to the biomimetic properties of the cellulose-based hydrogel.The 3D environment more closely mimics the native extracellular matrix, promoting cell-cell and cell-matrix interactions that influence mechanotransductive signaling (Yamada et al. 2022). This, in turn, may enhance the innate immüne response, including the upregulation of LL-37 (Ali et al. 2025). These findings sugest that the microenvironment plays a critical role in regulating MSC function and highlight the potential of 3D culture systems to improve their therapeutic efficacy. The increase in LL-37 transcription observed in 3D culture coincides with the findings of Chow et al., who confirmed the intracellular presence of LL-37 in MSCs after *S. aureus* challenge. All these results indicate that 3D-cultured PMSCs not only have intrinsic antibacterial properties but also increase the secretion of LL-37, highlighting their therapeutic potential in the treatment of bacterial infections. To our knowledge, this is the first study evaluating LL-37-associated antimicrobial responses of palatal MSCs within a cellulose-based 3D hydrogel system. Furthermore, the effect of PMSCs on biofilm formation and lysis assay was investigated using *P. aeruginosa* K6. It was observed that the experimental groups were not successful in lysing the biofilm formed by *P. aeruginosa* K6, while PMSCs-3D significantly inhibited biofilm formation, achieving an inhibition percentage of 57.65% compared to the control group (****P ≤ 0.0001). This finding is consistent with previous studies, reporting that MSC-conditioned medium inhibited biofilm formation by MRSA (USA300) (Chow et al. 2020).

While increased LL-37 expression was associated with enhanced antimicrobial activity in the 3D culture system, this relationship should be interpreted with caution. The present findings demonstrate a correlation rather than a direct causal link, as functional validation through inhibition or knockdown approaches was not performed. Therefore, further studies are needed to clarify the specific mechanistic contribution of LL-37 to the antibacterial effects of PMSCs.

## Conclusions

This study provides new insights into the remarkable antibacterial potential of PMSCs when cultured in a 3D hydrogel, demonstrating targeted activity against pathogen strains. Considering the limitations and adverse effects associated with traditional antibacterial therapies, such as resistance and toxicity, the use of PMSCs integrated into 3D cellulose-based hydrogels offers a convincing therapeutic alternative with safety and efficacy profiles. Nevertheless, further research is required to elucidate the specific molecular agents-particularly antimicrobial peptides-that mediate these antibacterial effects. If future research confirms these preliminary findings, this approach could conduce to the improvement of innovative and effective interventions for managing drug-resistant bacterial infections.

## Data Availability

Data supporting this study are included within the article.
